# Common strategic research agenda for radiation protection in medicine

**DOI:** 10.1007/s13244-016-0538-x

**Published:** 2017-02-15

**Authors:** European Association of Nuclear Medicine (EANM), European Federation of Organizations for Medical Physics (EFOMP), European Federation of Radiographer Societies (EFRS), European Society of Radiology (ESR), European Society for Radiotherapy and Oncology (ESTRO)

**Affiliations:** 1 EANM, Schmalzhofgasse 26, 1060 Vienna, Austria; 2 EFOMP, Fairmount House, 230 Tadcaster Road, York, YO24 1ES UK; 3 European Federation of Radiographer Societies, Catharijnesingel 73, Utrecht, 3511 GM The Netherlands; 4 ESR, Neutorgasse 9, 1010 Vienna, Austria; 5 ESTRO, Rue Martin V 40, 200 Brussels, Belgium

**Keywords:** Radiation protection, Research, Optimisation, Justification, Medicine, Dosimetry

## Abstract

**Abstract:**

Reflecting the change in funding strategies for European research projects, and the goal to jointly improve medical radiation protection through sustainable research efforts, five medical societies involved in the application of ionising radiation (European Association of Nuclear Medicine, EANM; European Federation of Organizations for Medical Physics. EFOMP; European Federation of Radiographer Societies, EFRS; European Society of Radiology, ESR; European Society for Radiotherapy and Oncology, ESTRO) have identified research areas of common interest and developed this first edition of the Common Strategic Research Agenda (SRA) for medical radiation protection.

The research topics considered necessary and most urgent for effective medical care and efficient in terms of radiation protection are summarised in five main themes:

1. Measurement and quantification in the field of medical applications of ionising radiation
2. Normal tissue reactions, radiation-induced morbidity and long-term health problems
3. Optimisation of radiation exposure and harmonisation of practices
4. Justification of the use of ionising radiation in medical practice
5. Infrastructures for quality assurance

The SRA is a living document; thus comments and suggestions by all stakeholders in medical radiation protection are welcome and will be dealt with by the European Alliance for Medical Radiation Protection Research (EURAMED) established by the above-mentioned societies.

***Main messages*:**

• *Overcome the fragmentation of medical radiation protection research in Europe*

• *Identify research areas of joint interest in the field of medical radiation protection*

• *Improve the use of ionising radiation in medicine*

• *Collect stakeholder feedback and seek consensus*

• *Emphasise importance of clinical translation and evaluation of research results*

## Preamble

Reflecting the changing funding strategies of research projects within Europe and the goal of jointly improving medical care by sustainable research efforts, the following medical societies involved in the application of ionising radiation, namely,

**European Association of Nuclear Medicine** (**EANM**)

The EANM is the umbrella organisation representing nuclear medicine in Europe and represents 40 National Member Societies, approximately 3200 individual members and around 30,000 professionals working in Nuclear Medicine in Europe. EANM aims to advance science and education in nuclear medicine for the benefit of public health, relating to the diagnosis, treatment, research and prevention of diseases through the use of unsealed radioactive substances and the properties of stable nuclides in medicine, throughout Europe.

**European Federation of Organisations for Medical Physics** (**EFOMP**)

The EFOMP serves as an umbrella organisation representing 35 national member and affiliated organisations of more than 7000 physicists and engineers working in the field of medical physics in Europe. EFOMP aims to harmonise and advance medical physic in both its professional clinical and scientific expression throughout Europe by bringing about and maintaining systematic exchange of professional and scientific information, through the formulation of common policies, and by promoting education and training programmes.

**European Federation of Radiographer Societies** (**EFRS**)

The EFRS is the non-profit umbrella organisation representing 39 professional societies and 51 educational institutions representing over 100,000 radiographers across Europe. The aims of the EFRS are to represent, promote and develop the profession of radiography in Europe, across medical imaging, nuclear medicine and radiotherapy areas of radiography practice.

**European Society of Radiology** (**ESR**)

The ESR is a non-profit organisation representing the general interests of radiology in Europe. The aims of ESR are to serve the healthcare needs of the general public through the support of science, teaching and research and the quality of service in the field of radiology as well as the promotion and coordination of the scientific, philanthropic, intellectual and professional activities of radiology in all European countries. The ESR has over 69,300 individual members as well as 59 institutional member societies of which 44 are national radiology societies and 15 are European Radiological Subspecialty Societies and European Allied Sciences Societies.

**European Society for Radiotherapy and Oncology** (**ESTRO**)

The ESTRO is a non-profit scientific organisation representing radiation oncologists, medical physicists, radiobiologists and radiation therapists with over 5000 members both within and outside Europe. ESTRO aims to foster the role of radiation oncology in order to improve patient care in the multimodality treatment of cancer by promoting innovation, research and dissemination of science through its congresses, special meetings, educational courses and publications.

decided that it was necessary and would be helpful to develop a corresponding common Medical Strategic Research Agenda (Medical SRA) to overcome current and future deficits and to be a constructive partner in European radiation protection research. To this end, research areas of interest have been jointly identified and agreed upon in this common SRA endorsed by the medical societies.

The effort of the medical societies in developing an SRA for the medical application of ionising radiation complements the efforts of other European platforms such as MELODI, EURADOS, ALLIANCE and NERIS, which have developed or are developing their own SRAs in the fields of general low-dose research, dosimetry, radioecology and emergency preparedness, respectively.

In a memorandum of understanding (MoU) signed by the medical societies, MELODI and EURADOS in 2014, it was decided to cooperate in order to promote the integration and the efficiency of European radiation protection research, to maintain and use a common European infrastructure for this research as well as to bring forward scientific education and training in the field of radiation protection for medical applications of ionising radiations.

The mission is to achieve the following objectives:

- Ensure an adequate level of information exchange between the signatories in the fields of joint interest within the scope of the MoU;
- Identify gaps of joint interest in existing SRAs with respect to RTD needs for improving radiation protection in the medical field, or for improving the effectiveness/exposure ratio of medical protocols based on the use of ionising radiations, so as to optimise the SRA contents and avoid duplication of efforts;
- Identify research areas of joint interest where progress may benefit from contributions from signatory organisations, or the members thereof, e.g. some low-dose effects or dosimetry research projects may benefit from contributions in a clinical environment, conversely, some medical protocol research may benefit from advanced dosimetry or radiobiology developments;
- Develop joint documents to support the elaboration of research and technological development (RTD) calls in the framework of the Horizon 2020 programme, both in the EURATOM/Fission and in the Health programmes;
- Optimise and coordinate the dissemination of scientific knowledge resulting from research, particularly through education and training actions.

The stakeholders are involved through a formal consultation process that has been initiated, is ongoing and will be reflected in future updates of the SRA presented here.

## Summary

Reflecting the change of funding strategies for research projects within Europe, and the goal of jointly improving medical care by sustainable research efforts, the medical societies involved in the application of ionising radiation have identified research areas of interest and agreed upon these in this common SRA endorsed by the medical societies.

The research that is seen to be necessary and most urgent for effective medical care, under the best harmonised practice, and efficient in terms of radiation protection can be summarised to the following five main topics:

1. Measurement and quantification in the field of medical applications of ionising radiation
2. Normal tissue reactions, radiation-induced morbidity and long-term health problems
3. Optimisation of radiation exposure and harmonisation of practices
4. Justification of the use of ionising radiation in medical practice
5. Infrastructures for quality assurance

The subtopics defined for each topic describe the specific research aspects that are identified as areas of great importance regarding research for establishing optimal radiation protection in the field of medical applications. These descriptions can be found in Chap. 3.

It is important to highlight that the approach to improve the use of ionising radiation in medicine by pure fundamental research would lack impact and influence unless having immediate consequences for and being translatable to everyday clinical practice. It is also important that the results of the research are not only translatable but really translated into daily routines. Therefore it is essential that the research is undertaken in a concise manner by persons educated and trained for good medical practice. The results have to be evaluated in clinical practice and have to be made public in a way that it is easy to access (results and implementation guidelines available on the internet) and to implement the methodologies developed. It is also essential that the same level of importance is placed on educating the staff working in the field to guarantee a direct clinical impact and to ensure high-level, standardised medical care and related radiation protection fully exploiting and profiting from all research conducted with regard to radiation protection in the medical field throughout Europe. This aspect of the SRA is reflected in Chap. 4.

## Background

Over the last 5 to 10 years the structure of research funding by the European Commission (EC) has gradually changed. The intention is to bring together all interested parties to facilitate European research projects in the field of radiation protection research and “*to set up a European umbrella structure for the administration of radiation protection research calls*”. To this end, SRAs have been developed or are currently under development.

Therefore, a medical SRA is especially important in view of the applications of ionising radiation in the medical field, since the medical use of ionising radiation is the largest man-made source of exposure to the human population. The advantages of such SRAs include:

- Providing guidance on/help to identify the most relevant and urgent research topics in the fields they cover
- Demonstrating the importance of research areas to the stakeholders
- Justifying research expenditure in defined areas
- Facilitating discussions with other members of the scientific community in the field of radiation protection
- Determining important topics and influencing research calls of the EC, OPERRA and CONCERT.

Since medical applications are among the most important contributors to exposure of the population in Europe to ionising radiation, for medical radiation protection research to be effective, it is critical that the results of the research projects are directly transferred into clinical practice, i.e. translational research.

This SRA has been the cornerstone for a common platform of the European medical societies dealing with topics related to the use of ionising radiation. In September 2016 the **European Alliance for Medical Radiation Protection Research** (**EURAMED**) was launched by EANM, EFOMP, EFRS, ESR and ESTRO and is currently run as a joint initiative under the umbrella of the European Institute for Biomedical Imaging Research (EIBIR). The medium-term goal is to establish EURAMED as a separate legal entity with a sustainable governacne and membership structure to allow other stakeholders to participate actively in the platform. Updates are available at www.euramed.eu.

## Research topics

### Measurement and quantification in the field of medical applications of ionising radiation

A key priority for radiation protection research in radiation oncology, nuclear medicine and also interventional and diagnostic applications of ionising radiation is to improve techniques and methods for measurement and quantification. The research approaches will need to be multidisciplinary and innovative. The key research questions in measurement and quantification research are:

#### Characterisation of exposure

The basic quantity for the characterisation of exposure is the absorbed dose, so whereever possible dose measurements or calculations/calibrations should be stated in terms of absorbed dose (1–3). One of the main challenges for future research is the pronounced anatomical heterogeneity of (absorbed) doses within and between critical organs in all areas of medical uses of radiation. This needs to be supplemented by optimisation of models and model parameters to translate absorbed doses into equivalent, organ, biologically effective doses or any other indirect dose entities. Accurate and precise measurements with known uncertainty (4, ) are a prerequisite for the adequate implementation of dosimetric techniques into medical practice and medical routines, specifically for different types (qualities) of radiation and levels of spatial resolution. Therefore, the following issues need to be addressed in research:

- Calibration of dosimeters for medical applications is currently performed using secondary standards non-specific to the radiation fields used in medical application of ionising radiation leading to undefined measurement uncertainties. Therefore, exact measurements require calibration against radiation fields specific to medical applications.
- There is a limited availability of dosimeters for use inside the human body; this implies that currently simulations of radiation transport and deposition are necessary, e.g. using Monte-Carlo (MC) methods (6, 7), as is normalising them to measured quantities.
- Real-time measurement of doses is relevant to reduce doses to staff. Therefore, the development of specific dosimeters is required, allowing real-time monitoring, e.g. of eye structures and extremity/finger doses, from interventional radiology/cardiology and nuclear medicine. The existing dosimeters are either not for online measurements or they suffer from technological limitations in terms of highest dose rates as in pulsed radiation fields or size or practicability.
- Non-uniform spatial (3D) and temporarily varying (4D) dose distributions can lead to differences of up to several orders of magnitude in local dose distributions (8). Therefore, micro-dosimetric measurement devices and techniques for use within and between cells, the anatomical structures of organs and the human body are necessary, e.g. for dosimetric use with regard to individual structures in the eye, the brain and the heart, and also other organs depending on the basis of future research results.
- Different types of radiation (photons, electrons, protons, heavy ions, secondary neutrons) are used for and/or associated with medical purposes. Correct determination of doses to and dose-distributions within patients at different levels of spatial resolution is necessary depending on the required purpose in terms of radiobiological questions or optimisation of procedures. Also mixed fields and energy spectra need to be taken into account for reliable measurements and calculations of dose-distributions.
- Knowledge on track structure and/or microdosimetry of internal emitters (alpha, beta, Auger) is a prerequisite to predict the associated biological effects (9). Therefore, computational methods need to be further developed and connected to the results of corresponding research on measurements and calibration procedures (see above).
- Development of updated or alternative quantities and concepts for describing the anatomical dose distributions within organs, tissues and the body as the basis for predicting health effects rather than mean absorbed doses (e.g. dose averaged over an organ) or dose volume histograms.
- Methodologies have to be developed for determination, description measurement and calculation of doses outside the planning target volume (PTV) for radiation therapy, i.e. the peripheral dose. This is urgently required to build and optimise prediction models for secondary tumours, but also tissue effects, and to enable comparison of different techniques and/or technologies.

This research would be a prerequisite for the accurate and precise evaluation of the dose as the basis for better radiation protection of the patient and medical personnel as explained below.

#### Individual dosimetry

Individualised patient dose assessment methods, e.g. by adjusted phantoms for measurements (10), size-specific conversion factors, dose measurements taking into account imaging parameters shielding, etc., are needed to allow for accurate patient dose estimation (2) and risk assessment (11). Many dose distributions would depend on individual patient constitution (e.g. size, weight, shape, age and biological factors such as the distribution and kinetics of radioactive markers () or susceptibility to different therapeutic procedures). Therefore, the following dosimetric procedures need to be addressed in research:

- Development of computational methods for dose distribution calculations based on patient-specific and equipment-specific characteristics for all medical procedures using ionising radiation, including for example CT, interventional and nuclear medicine procedures as well as radiotherapeutic procedures avoiding different dose indicators for different types of procedures in order to get comparable meaningful information about organ doses of individuals.
- Development of optimal measurement protocols in nuclear medicine for accurate estimation of absorbed doses using patient-specific and equipment-specific characteristics. Refinement, validation and implementation of new biokinetic models for dosimetry in molecular radiotherapy using for example physiologically based pharmacokinetic (PBPK) models for the individual assessment of biokinetics (13), including uncertainty budgets (14).
- Development of methods to estimate or measure the actual delivered radiation dose in radiotherapy.
- Development of a unique dose indicator that describes the absorbed dose to organs in order to perform risk assessment.

This research would be essential for accurate and precise determination and evaluation of indication-, therapy- and/or subgroup-specific doses and therefore risks of radiation-induced morbidities of individual patients and thus on a per-patient basis for better radiation protection of patients and medical personnel.

#### Quality metrics for diagnostic imaging and therapy

For the use of quantitative imaging approaches, standardised protocols for each clinical indication and/or specific disease common clinical indication need to be developed (15). Therefore, the following issues need to be addressed in research:

- Development of dosimetric and image quality metrics to fully assess the impact of novel detector technologies (e.g. low or lowest noise as well as energy-resolving detectors) and image reconstruction methods available for reducing radiation exposure to the patients. To this end, research is needed on which requirements (system stability, noise reduction, influence of individual patient characteristics, iterative reconstruction parameters) have to be met for quantitative imaging to yield reliable and reproducible results.
- Measuring methods (e.g. phantoms, reading protocols, etc.) need to be improved or developed and standardised to address the improvements in medical technology as well as new methods, e.g. particle therapy or new molecular imaging technologies.
- There is an increasing need also for quality metrics of treatment plans to allow easier quality assurance to facilitate comparability of methods used in radiation therapy and to allow more standardised research regarding clinical treatment outcomes.
- The concepts and the use of diagnostic reference levels (DRLs) and achievable dose levels (ADLs) have to be redefined to meet the requirements of organ-specific dose distributions or critical organ structures doses.

This research enables the translation of quantitative techniques to widespread clinical use for the benefit of the patient. In addition, this research is also a prerequisite for the harmonisation of practices and quality assurance.

#### Sources and influences of uncertainty

Uncertainties need to be determined for all techniques described above, be it measurements or computations. Many components independently contribute to the uncertainty in the determination, reporting and performance of medical applications and in its characterisation (4, 16). It is of utmost importance to develop methods to assess the contributions of different stages in the chain of medical interventions to be able to define the relevant points of optimisation, which means putting effort into those parts of a medical application scheme where there is the highest benefit. Therefore, the following issues need to be addressed in research:

- Quantification of the influence and sensitivity of different parameters (technique dependent, system dependent, patient dependent, medical staff dependent).
- Development of methodologies for classifying different influencing parameters and to build a system that allows the optimisation of medical applications of ionising radiation for individual patients or methods.

Knowledge of the integral uncertainty and its components is key to identifying the most relevant steps, to allow for prioritisation and targeted optimisation, thus making more effective use of clinical and research resources.

### Normal tissue reactions, radiation-induced morbidity and long-term health problems

A key priority for radiation protection research in radiation oncology, nuclear medicine and also interventional and diagnostic applications of ionising radiation is to improve health risk estimates. The corresponding research approaches will need to be multidisciplinary and innovative. The key research questions in tissue reactions and biological risk research are:

#### Exposure-associated cancer risk: dose, dose distribution and dose-rate dependence

Knowledge of the dose dependence of the radiation induction of primary or secondary cancers, in particular in relation to dose inhomogeneities and dose rate, is of major importance to optimise therapeutic efficiency and reduce unwanted side effects. In radiation oncology, this refers to high doses within the planning target volume (PTV) as well as to out-of-PTV doses, e.g. low to moderate doses, in particular in intensity-modulated and image-guided radiotherapy, but also in brachytherapy and molecular (radionuclide) radiotherapy (17). It also needs to include other, additional treatment modalities, particularly chemo- and biologically targeted therapy. Diagnostic procedures must also be considered, especially in view of interventional or fluoroscopic procedures or nuclear medical imaging techniques and those applied in preparation for treatment.

#### Non-cancer effects in various tissues and radiobiology-based effect models for individual morbidity endpoints

Radiation-induced morbidity (cancer and non-cancer diseases and disorders) may be observed early or late (occurring after 3 months to 5 years after radiation exposure), not only in the tissues and organs exposed to high doses. Also, very late health effects (occurring after more than 5 years to many decades after exposure) may not only be observed in high-dose radiotherapy (>5 up to 50 Gy) but also in the intermediate (0.5 to 5 Gy) or low-dose (<0.5 Gy) ranges. Examples of these very late occurring normal tissue morbidities, which may be induced by localised radiation exposure outside the planning target volume of radiotherapy or by repeated interventional procedures, are: cardiovascular or cerebrovascular diseases, functional or structural damage to eye structures, various delayed, persistent immunological changes, progressive microvascular injuries, but also late and very late developmental and functional detriments after radiation exposures in diagnostic procedures and paediatric radiotherapy and many more radiation-associated health disorders. The contribution of other treatment modalities, particularly chemo- and biologically targeted therapy, to the development of very late side effects is currently poorly understood and needs also to be considered along with any diagnostic procedures, especially for interventional or fluoroscopic and nuclear medicine procedures and those applied in preparation for treatment.

Current morbidity risk models and normal tissue complication probability (NTCP) models are largely empirical or based on hypothetical data-fitting models of assumed processes of damage development and lack the evidence of a mechanistic basis. Moreover, they do not consider the influence of the position of the doses within one organ or the interaction of dose distributions in “corresponding” organs, such as lung and heart, or the effect of additional treatments, such as chemotherapy (18, 19). These factors, however, must be included to get appropriate estimates for the patterns of risk of any individual patient with regard to modern techniques in radiotherapy, nuclear medicine and radiological diagnosis.

#### Individual patient-related radiation sensitivity and early biomarkers of response and morbidity

The individual sensitivity of patients may be considered in the choice of specific diagnostic procedures and/or therapeutic strategies. This can be based on intrinsic factors (age, gender, genomics, proteomics) of their tumours or different normal tissues, but also on concomitant diseases impacting on general or specific normal tissue tolerance, lifestyle (e.g. reduced lung/liver tolerance due to smoking and alcohol consumption) or previous/parallel treatments.

In a number of tumours, biological factors affecting radiosensitivity, i.e. predictive factors, such as local hypoxia, tumour heterogeneity, or viral infections, were identified. Such investigations need to be extended and may also consider the early response of the tumour to a specific treatment. Imaging biomarkers of tumour radiosensitivity are needed in this context, as well as biomarkers of morbidity, which can be identified before or early in the treatment phase and may help in the selection of the adequate treatment of the individual patient. These have so far been rarely studied. However, patients with a high risk for a certain, severe, morbidity symptom may require a change in dose distribution or in treatment strategy, or follow-up protocols may need to be adjusted to the individual morbidity risk pattern based on early biomarker expression ().

#### Radiobiological mechanism of radiation-induced side effects and protective strategies

The radiobiological molecular mechanisms of radiation-induced morbidities in normal tissues and organs are very complex and vary between different signs and symptoms of morbidity in the same organ and between different organs. Also the tumour responses to therapeutic exposure to ionising radiation, including radiotherapy using hadrons, are currently largely unknown. The radiobiological molecular mechanisms are even more complex for combined radiotherapy and chemo- or biologically targeted treatment strategies. These mechanisms need to be clarified for specific clinical morbidity endpoints in order to develop specific strategies for protection, mitigation or management of the clinical consequences of exposure. They are even more important for medical radiation procedures in paediatric patients given the evidence showing that the complexity and severity of morbidities and developmental injury and the risks of therapy-induced malignant diseases are particularly high after radiotherapy (in almost all instances in combination with chemotherapy).

Similarly, novel strategies for improving the diagnostic and/or therapeutic efficacy for the application of ionising radiation may be based on the synergistic combination with upcoming technologies such as combinations with high-intensity focussed ultrasound and biology-based approaches relying on tumour genomics, proteomics or metabolomics including local enhancement of drug delivery.

Both the protective and sensitising strategies need to be established and validated in preclinical as well as in subsequent clinical studies. These investigations need to focus on the efficacy of the novel approaches and also on their selectivity for the respective target tissue to guarantee a therapeutic gain.

### Optimisation of radiation exposure and harmonisation of practices

According to the European Basic Safety Standard (BSS) (2013/59/EURATOM) (21), the radiation protection of individuals subject to public or occupational exposure must be optimised with the aim of keeping the magnitude of individual doses, the likelihood of exposure and the number of individuals exposed as low as reasonably achievable (ALARA) taking into account the current state of technical knowledge, economic and societal factors. The optimisation of the protection of individuals subject to medical exposure should be consistent with the medical purpose of the exposure.

The EU Directive on patients’ rights in cross-border healthcare (2011/24/EU) (22) calls for a concerted strategy in terms of harmonisation of clinical practices, meeting patients’ expectations of the highest quality healthcare, including when they seek treatment away from home.

According to the literature, high variability of mean effective doses or organ doses of patients across Europe persists across all medical ionising radiation procedures and is seen across single countries, hospitals or even at the departmental level (23), despite technological developments facilitating reductions in patient dose, thus highlighting the importance of harmonisation of ionising radiation procedures and the development of new and more efficient optimisation methods including evaluation criteria. For this optimisation, there needs to be a general definition as to what is an acceptable level of quality, what kind of optimisation should be performed and what is the optimal level. With the main goal of maximising the clinical outputs of the procedures while minimising the exposure of patients and staff, the key research questions are:

#### Patient-tailored diagnosis and treatment

The comprehensive tailoring of imaging and therapeutic procedures in terms of the clinical question, anthropometric and physiological parameters of each patient, especially children, and lesion-specific characteristics is a key challenge that is largely yet to be fully addressed. Furthermore, imaging is essential to patient-tailored therapy planning, therapy monitoring and follow-up of disease, as well as targeting non-invasive or minimally invasive treatments, especially with the rise of theranostics (combination of diagnostic and therapeutic procedures to optimise treatment).

For the reasons given above, and in view of reducing radiation exposure to the patients by individually tailoring their diagnosis and treatment, research needs to be conducted with regard to the following currently unresolved issues:

- Development of quantitative imaging biomarkers for each common clinical indication and/or specific disease/organ and their standardisation with regard to required image quality in conjunction with related radiation exposure.
- Recent advances in imaging using specific radiotracers will provide additional tools for better characterisation of a lesion at the molecular level. This will provide an insight into lesion heterogeneity and targeting, with perspectives in guiding biopsy of lesions, prediction of treatment response and image-guided therapy.
- For optimal treatment prescription in targeted radiotherapy the knowledge of the dose-response relationship is essential. In targeted radiotherapy, patient-specific dosimetry is essential for both the prediction of the adverse events of a treatment and of the tumour response (24).
- Research on the requirements that have to be met for quantitative imaging to yield reliable and reproducible results, e.g. in view of system stability, image reconstruction techniques, influence of individual patient characteristics and applied radiation exposure.
- Development of approaches for low-dose time-resolved volumetric imaging (4D), e.g. of blood flow or volume distribution (perfusion) as well as organ-motion dependent imaging, especially in view of therapy planning and treatment response imaging.
- Development of body-mass index (BMI)-specific image acquisition protocols and specific dose-reduction algorithms for obese patients, since obese patients require higher than average radiation doses, and exploitation of techniques normally used for radiation exposure reduction to achieve diagnostic image quality.
- Development of approaches for low-dose treatment response and follow-up imaging solely focussing on the detection of “change” (relative to a standardised baseline acquired at higher radiation exposure) providing reliable diagnostic assessment, e.g. through development of standardised disease- or treatment-specific imaging protocols especially for those patients frequently imaged.
- Research for identifying underlying relationships among demographic, disease-related and ‘omics’ biodata and image and treatment data for fully developing personalised medicine in order to offer the best medical diagnostics and treatment associated with the lowest possible dose to each individual patient.

The benefit of this research could be to develop systems for diagnosis and treatment allowing for more efficient treatment techniques, which may also offer economic benefits. This research could also provide further insights into disease processes of individual patients and therefore foster precision medicine.

#### Full exploitation and improvement of technology and techniques

Despite the potential for the exponential growth in the technological features of medical imaging equipment to decrease patient doses, such benefits are not always realised in daily clinical practice (25).

Therefore research on development, improvement, clinical applicability and full clinical exploitation of (new) technology and techniques for offering diagnosis and treatment delivery associated with the lowest technically possible radiation exposure to the patients is required. In this context, currently the following topics need to be addressed by research:

- Low-dose CT imaging enabled by low tube potentials and current-time products in view of its clinical applicability, indication, standardisation as well as its potential diagnostic and technical limitations.
- Novel image reconstruction techniques enabling low- or lowest-dose image acquisitions, with regard to their routine clinical applicability and their limitations in view of ensuring diagnostic accuracy and reliability.
- Novel detector technology in medical imaging in view of its clinical applicability and potentially associated technical limitations.
- Diffraction enhanced imaging and other newly developed approaches.
- Further development, implementation and application of patient- and disease-adapted techniques and protocols of combined modalities as for example SPECT/CT (26), PET/CT, PET/MRI and LINAC-MRI.
- Optimisation of image guidance procedures in radiotherapy.
- Strategies for a reduction in peripheral doses in radiotherapy, e.g. by defining indications for ion therapy.
- Research for, and production of, novel radionuclides and radiopharmaceuticals for either improving diagnostic and therapeutic outcome or reducing associated exposure.
- Data-crawling and -mining approaches based on large-scale data contained in imaging and treatment biobanks, e.g. for extracting indication-specific acquisition or treatment protocol parameters along with associated patient exposure data for the purposes of diagnosis and treatment optimisation, standardisation and harmonisation (through the definition of European DRLs) as well as for extraction of higher-order patterns of disease, its diagnostics and treatment along with associated doses, and the possible interrelation of this data, e.g. to genomic data (radiogenomics).

While research with regard to technology development may remain basic research that is institution- or manufacturer-driven and controlled, though requiring and relying on input and feedback from medical research and routine clinical applications, research on clinical applicability, improvement and full exploitation of technology and techniques enabling radiation exposure reduction is driven by, and requires, active medical research in the fields of radiological diagnosis and radiopharmaceutical and therapeutic treatment. There needs to be an emphasis on the close link between technology developments at research institutions, especially at manufacturers’ sides, and the clinical research facilities with feedback options and especially to define a process to consolidate the achievements in terms of harmonisation.

Any optimisation in medical imaging techniques, including dose reduction strategies, must be evaluated thoroughly in terms of the resulting image quality. In determining whether an image is diagnostic or fit for purpose, it is important to take into account not only the physical measurements of image quality [e.g. signal-to-noise ratio (SNR), modulation transfer function (MTF) and detector quantum efficiency (DQE)] but also to include psychophysical methods (e.g. contrast detail assessment and spatial resolution assessment) and clinical, diagnostic performance approaches such as visual grading analysis (VGA), receiver-operating characteristic (ROC) and psychometric scales. The current variability and absence of validated approaches and guidelines represent a significant barrier to effective optimisation research. The 1996 European Guidelines on Quality Criteria for Diagnostic Radiographic Images (27) aimed to provide some assistance with image quality assessment but these were very limited, have deficiencies, were never validated and are now dated. There is thus an urgent need for establishment of robust, validated approaches to facilitate this critical aspect of optimisation research.

Technologically meaningful developments, with respect to the possible output for patient, staff and public, are at varying levels of maturity in terms of a technologies status as a product line and their applications in the medical environment.

In this context, multi-professional engagement together with educational institutions and equipment manufacturers will facilitate the required development of strategies for the harmonisation of ionising radiation procedures and standards of practice, since several studies have highlighted the heterogeneous use of technology and the unanticipated patient and staff dose increases. This is of particular importance in paediatric populations as well as for patient cohorts requiring multiple consecutive diagnostic, radiopharmaceutical or therapeutic procedures.

#### Clinical and dose structured reporting

##### Clinical reporting:

Medical imaging procedure workflow involves several steps, ending with a clinical report. Currently, medical imaging reports are often presented with little or no structure to the text. This can present difficulties in understanding the content of the report for both referring physicians and patients. The development of a structured reporting system will improve the clinical outcome of a medical imaging procedure, by focussing on the essential message, in a harmonised way, thus facilitating the communication process along the clinical pathway of the patient.

There are many advantages of such reports, including improved follow-up for returning or chronic patients, easy retrieval of pertinent information enabling clinical and translational research, integration of the information in imaging biobanks and automated translation.

Another related issue is the lack of a centralised medical databank on imaging procedures for each individual patient on a national and European level, often leading to unnecessary repeated diagnostic procedures and hence unnecessary radiation exposure. Harmonisation of clinical reports could facilitate the development of such a centralised medical registry at a European level. Also, a centralised dose data collection algorithm for therapeutic procedures would allow for improved analyses of dose-effect relationships for adverse events, including stochastic radiation sequelae.

##### Dose reporting:

Structured dose reporting in radiation diagnostics and therapy (or documentation of administered activities in nuclear medicine) is a growing area of focus and will benefit all professions directly involved in the ionising radiation procedures and patients undergoing such procedures in the years to come. However, the adequate specification of dose distributions has not been addressed yet in research and clinical practice sufficiently (1). In radiation oncology structured dose reporting needs to address absorbed doses in organs at risk and/or at their subvolumes, relevant for adverse event endpoints. The latter needs to be specified and their scaling to be defined. Moreover, anatomy-related dose distributions in the irradiated volume and in the periphery, at least down to the 1% isodose, need to be reported or re-constructible from the documented treatment information and then specifically related to potential radiation sequelae.

The main benefits would be:

- To establish a model for providing information, in radiation diagnostics and nuclear medicine, about patient dose exposure in an easily accessible way (e.g. by integrating visual scales for the referring physicians to understand the level of exposure).
- To facilitate the rapid determination of local, national and European DRLs.
- To facilitate establishment, in radiation oncology, of dose response relationships for adverse events in organs at risk as well as for stochastic radiation effects both close to the PTV and in the periphery of the patient.

Structured dose reporting in radiation diagnostics (or documentation of administered activities in nuclear medicine) is an essential tool for the harmonisation of the dose management systems and the comparison of doses, creating a comprehensive, common language for health professionals. Structured dose reporting in radiotherapy is essential to establish firm dose-effect relationships for adverse deterministic and stochastic events.

#### Protection of staff, patients, carers and the general public

Aside from the optimisation of protocols and procedures, their standardisation and their personalisation, it is most important to optimise radiation protection using existing radiation protection measures (28). To optimise radiation protection in terms of applicability and best benefit for staff and patients, the establishment of key indicators of safety and quality in radiation protection is essential according to the general ALARA principle discussed before. The primary goal of the development of safety programmes is to reduce morbidity risks from excessive exposure to ionising radiation for specific procedures and populations, e.g. interventional radiology and the paediatric population. Another focus is on cost-benefit analysis of the implementation of radiation protection devices and safety programmes. Neither proven criteria of cost nor proven criteria of benefit have been established so far. Research must explore both external and internal radiation exposure and their associated protection measures.

### Justification of the use of ionising radiation in medical practice

The principle of justification is one of the key pillars of radiation protection underlined in the recently revised European BSS Directive (21). This principle focusses on weighing the benefits versus the risks. Further important elements are patient communication, as the basis for shared decision-making including the patient rights for influencing the decision, as well as the appropriateness of the radiological procedure with respect to the clinical setting. The key research questions in research into the justification of the use of ionising radiation in medical practice are:

#### Benefit/risk assessment and communication

While the clinical benefit of a diagnostic or interventional imaging procedure is assumed to be established, an estimation of the risk related to effective dose exposure for a given patient is a difficult step because the current estimations are for a general population. The current uncertainties in this area make the establishment of a reliable benefit/risk assessment virtually impossible.

Therefore there is the urgent need for research aimed at risk estimation for an individual patient. However, it is unclear how this can be implemented for the stochastic mechanisms based on epidemiologic data. Increased risk factors for organ-specific patient groups or patient-parameter-based changes on optimal imaging procedure setups may however be investigated. For the development of such a research programme for diagnostic imaging and interventional procedures, reference to a centralised repository of imaging data would be an important resource for data mining and the following risk assessment (see Sects. 3.5.1 and 3.5.2).

The proposed research will have a direct benefit for the patient in general and especially in the context of screening methods based on the use of ionising radiation.

Most new therapeutic radiation technologies are clinically introduced to reduce exposure to healthy tissue. In the near future, an increasing number of cancer patients will be treated with particles (e.g. protons and carbon ions). Although particle therapy will result in lower dose levels to many critical structures as compared to the currently used photon-based technologies, the consequences in terms of reduction of late and very late side effects remain to be determined and have to be weighed against the higher costs.

In the context of the current drive for patient empowerment and involvement in the decision-making process, the development and subsequent evaluation of novel tools for patient communication have become necessary. Some professional organisations such as the ACR, ESR, RSNA and national clinical societies have developed communication guidelines and platforms for diagnostic imaging; however, a unified approach regarding methodology and content is currently missing.

The proposed research work will aim to develop a European evidence-based electronic communication platform focussing on all types of diagnostic imaging using current information technology that is endorsed by the relevant professional organisations, patient organisations and other relevant stakeholders. The European platform will be designed in a way to allow for localisation and adaptation to the national/regional settings. The establishment of such a system has to be based on the successful completion of the cost-benefit research activities outlined above.

#### Improvement of use of evidence-based guidelines

Clinical imaging guidelines are intended to help physicians decide when an imaging study would be useful and identify the most appropriate examination for a particular patient. In recent years, imaging guidelines, in view of the referral process, have received much attention from the radiation protection community and international organisations given the increasing number of medical imaging procedures and studies that have shown that about 30% of the imaging procedures performed in Europe were found to be inappropriate (29). The recently revised European BSS Directive (27) requires that clinical imaging guidelines are available in all EU Member States.

In 2011, the European Commission awarded a European tender project to assess the availability and implementation of clinical imaging guidelines in EU member states. One of the key conclusions, also highlighted in subsequent studies, was the recommendation that the awareness and use of clinical imaging guidelines in Europe need to be improved and novel approaches are needed for that purpose (30).

The proposed research work should identify and develop methods to improve the use of clinical imaging guidelines in Europe especially in view of the referral process at large, e.g. through incentives, regulatory requirements, IT tools, etc. The research work is related to a key priority in medical radiation protection as outlined among others in the Bonn Call for Action (31) and must be relevant for all diagnostic applications of ionising radiation. To define the proposed methods, an evaluation and impact assessment of the use of currently existing European and national guidelines must be performed with an emphasis on evaluating the usability of the guidelines and their impact on daily clinical practice (29, 32).

The outcome of the proposed research work should be a European recommendation paper on how to improve the dissemination, integration into the clinical workflow and use at large of clinical imaging guidelines in view of the referral process. In addition methodologies and guidelines for adoption/localisation/adaptation of the guidelines need to be proposed.

The recommendation paper shall serve as guidance for professional societies and policy-makers in Europe.

### Infrastructure for quality assurance

To perform investigations on tissue reactions, optimisation procedures as well as risk and benefit evaluations, it is important to rely on optimal, quality assured data, which are gathered under defined conditions and which are necessary for various reasons including legal questions pertaining or specific to the research to be performed. In addition, the clinical system of medical applications of ionising radiation has to be standardised (33) and evaluated concerning its effectiveness in radiation protection.

#### Data coding, collection and management

It is crucial for the future of medical imaging in Europe to develop a European medical imaging coding system (EMICS) including radiology and nuclear medicine imaging procedures. EMICS should apply to all medical procedures based on ionising radiation, giving policy makers and healthcare providers an objective and clear view, on a procedure-level basis, at the national and EU levels. This would be a fundamental tool for future studies such as population dose studies and/or parameter-dependent image quality studies. According to the recently published Dose DataMed 2 report “*in order to compare x-ray examination frequency data between countries*, *and to assign typical effective dose values to examinations*, *it is crucial that an* ‘*X-ray examination*’ *is defined and counted in a consistent way*” (34). Therefore, the development of EMICS, based on an alphanumerical code structure, must be facilitated and must be integrated into all HIS/RIS systems.

EMICS would also support the harmonisation of the “language” for medical imaging and therapy across Europe giving healthcare providers a powerful tool for the future planning of health systems at local, regional, national and European levels. This should be extended to the acquisition of data on the long-term consequences of radiation exposure, diagnostic or therapeutic, potentially in combination with other therapeutic procedures, to allow structured long-term follow-up, assessment and documentation of treatment-related morbidity and the possibility to relate morbidity to anatomical dose distribution. Requirements and structures, along with administrative characteristics, including data protection issues, need to be defined. Such data management structures will provide a basis for epidemiological investigations into relevant medical questions. Data should be collected throughout Europe according to this standard using defined mandatory and where possible additional data regarding exposure and if possible image quality as well as certain patient-specific data.

#### Comprehensive medical database/imaging biobank

Biobanks are repositories for the storage and retrieval of biological samples of a large number of subjects. A major goal of biobanks is the organised collection of biological material and associated information to spread access among scientists requiring this information. Extending this concept to medical imaging and especially to radiation protection is needed to collect radiation protection metrics and to allow for long-term follow-up for specific cohorts, which will be called a comprehensive medical database or imaging biobank. It might be important for various reasons:

##### Importance for dose collection:

The concepts and the use of DRLs and achievable dose levels (ADLs) have to be redefined to meet the requirements of organ-specific dose distributions or critical organ structure doses as mentioned in Sect. 3.1. Large-scale (national, regional) patient inter- and intra-organ dose distribution monitoring is necessary for the purpose of definition, optimisation and periodic assessment of DRLs and ADLs. This aim can be achieved by developing large-scale archives and automatic data analysis using the recently developed standards allowing sending and archiving of dose information.

The development of automatic methods for phantom image quality assessment (and patient image quality assessment) together with the use of advanced IT technologies (e.g. large-scale archives, data-mining methods, expert system technique) is required for supporting users in the optimisation process.

##### Importance for long-term follow-up of cohorts:

There is clear evidence that radiotherapy may cause, in organs and tissues close to the PTV but also in organs in the periphery, an increased risk for late and very late side effects that are clinically relevant and have a major impact on quality of life. Although there is an increasing awareness of radiation-induced very late side effects, the infrastructure to systematically collect relevant data to get more insight in the factors that contribute to these risks is largely lacking.

The proposed research work should involve the development of a structure for a European imaging biobank infrastructure integrated with a European radiation oncology biobank infrastructure.

#### Developing key performance indicators for quality and safety

Key performance indicators (KPIs) have been successfully introduced as a performance measurement in many areas of healthcare in line with the EU Agenda on Quality of Health Care and Patient Safety put forward by the EC DG SANTE. Currently there is no recognised gold standard in the fields of medical imaging or radiation therapy. A general concept of performance indicators for imaging and radiation therapy is thus needed and should also include indicators for the safety of patients and of procedures and how to maintain safety standards, according to the optimisation and justification processes.

The proposed research work will consist in the establishment of KPIs for the quality achieved regarding specific medical procedures and in general terms of radiation protection and harmonisation at the European level. For integration into the workflow, pilot studies in dedicated centres and impact assessment before dissemination are envisaged.

#### Audit systems

Clinical audit is a tool designed to improve the quality of patient care, experience and outcome through formal review of systems, pathways and outcome of care against defined standards, and the implementation of change based on the results. Audit cannot be carried out without a preset standard against which performance can be assessed.

As laid down in the revised European BSS Directive (21), Member States shall ensure that clinical audits are carried out in accordance with national procedures. Clinical audit is a relatively new concept in radiation protection. It seeks to improve the quality and outcome of patient care through structured review of medical radiological practices, procedures and results, whereby these are examined against agreed standards for good medical radiological procedures, with modification of practices, where appropriate, and the application of new standards if necessary.

In October 2009, the EC published guidelines relating to clinical audits for radiological practice, including all investigations and therapies involving ionising radiation (35). In spite of this document, clinical audit is still clearly underdeveloped in Europe. To address this shortcoming, the proposed research must aim to develop an easy-to-use, cost- and time-effective European clinical audit tool taking into account existing initiatives from professional organisations. The tool will facilitate implementation of the relevant requirements in the European BSS Directive and could potentially provide the basis for future European accreditation processes based on quality and safety.

#### Education and training metrics

There is a strong demand for new education and training models in medical radiation protection because of the rapid development of medical techniques based on ionising radiation, growth of hospitals and the continuous need to produce competent health professionals. The major challenge is addressing the variety of professions and professionals, with different knowledge background and different needs, but all working towards the same objective: patient and staff safety (36, 37).

To achieve that objective it is necessary to establish a harmonised and sustainable safety culture in radiation protection amongst health professionals through specifically oriented education and training courses. External assessment of the quality of education or training provision is needed (37) and should be provided by a European accreditation body.

It is important to develop through research:

- A metric system to measure the knowledge, skills and competence outcomes from education and training in radiation protection for the different health professions involved in ionising radiation procedures.
- An assessment system to measure:
  - the impact of the implementation of a continuous professional development model for education and training in radiation protection;
  - the type of needs for education and training, considering the installation of new equipment and/or new procedures.

There is a need to create a European certification system for education and training in radiation protection, based on the development of standards of proficiency for health professionals, as an instrument to guarantee safety procedures to European citizens, through harmonisation of practice through education and training.

## Education and training

As highlighted in the recent EC Radiation Protection No. 175 ‘*Guidelines on radiation protection education and training of medical professionals in the European Union*’ there is a continuing and growing need for high-quality education and training in the field to ensure the radiation protection of patients, staff and the public. This education and training must be accessible and delivered at an appropriate level for all professionals working in the field of medical ionising radiation as well as those utilising the services provided by medical ionising radiation professionals. EC Radiation Protection No. 175 came about as an outcome of the MEDRAPET project and describes education and training in radiation protection using the European qualifications framework (EQF), knowledge, skills and competence (KSC) structure and European credit transfer system (ECTS) (38).

It is essential that any research in the area of medical ionising radiation is translated into clinical practice to ensure that patients and staff see the direct benefits of this research. As highlighted in Sects. 3.3 and 3.4 of this SRA, there is evidence that this translational research often fails because of the absence of parallel education and training programmes. High-quality education and training programmes will raise awareness of ongoing EU research projects and initiatives and ensure their uptake into clinical practice at local, national and European levels. Separately, there has been an identified need to also develop high-quality education and training specifically for researchers to help strengthen the medical ionising radiation research community.

Education and training may consist of traditional, face-to-face lectures and practical sessions but should also focus on becoming more clinically focussed and case based. Online, or e-learning, approaches to the delivery of content at all levels utilising mobile devices is a key consideration, which includes the development of dedicated appropriate e-learning tools, e.g. facilitated by a multidisciplinary European e-learning platform.

### Education of staff

In the former chapters necessary and relevant topics for research related to the optimal use of ionising radiation and radiation protection in medical applications have been explained. Also, measures have been mentioned concerning how these optimisation have to be implemented throughout European by means of standardisation and harmonisation. However, it is obviously not sufficient just to define methods for harmonisation but this has to be reflected within the education of the staff (28, 39).

This education needs to reflect the basic aspects of:

- radiation physics,
- radiation biology,
- radiation protection,
- radiation communication and
- specific parts for the procedures/areas that are supposed to be covered by the staff.

Therefore, within this SRA it is proposed to develop a standardised education rule describing topics that have to be covered. In addition there is a need for securing the highest level of knowledge transported reflecting state-of-the art technology as well as standardisation and harmonisation efforts. Finally, establishment of a European certification approved by the medical societies issuing this SRA should also be covered, not only after the completion of initial training, but also throughout the whole professional life of each professional.

### Education of researchers

To provide valuable research dealing with these identified relevant topics with potential impact, it is important to perform well-founded and structured research along certain lines. To do so, it is also necessary to train researchers in performing research according to the best practice. This especially holds true for research working with humans or biological material, but also with any data related to humans. There has to be a standardised training structure also reflecting the actual state of the art for research procedures with the goal of fostering the efficiency of projects reflecting the research topics identified above especially in terms of optimal patient care and radiation protection.

In this respect it is important to deal with best practice regarding:

- literature and citation practices;
- statistical power of investigations;
- uncertainty budget calculation of measurements and calculations/simulations;
- clear hypothesis-driven project definition;
- pre-research feasibility estimates of proposed outcomes.

ACR, American College of Radiology; ADLs, Achievable Dose Levels; ALARA, As Low As Reasonably Achievable; ALLIANCE, European Radioecology Alliance; BMI, Body-Mass Index; BSS, Basic Safety Standard; CT, Computed Tomography; CONCERT, European Joint Programme for the Integration of Radiation Protection Research; DE, Dual-Energy; DRLs, Diagnostic Reference Levels; EANM, European Association of Nuclear Medicine; EC, European Commission; ECTS, European Credit Transfer System; EFOMP, European Federation of Organisations in Medical Physics; EFRS, European Federation of Radiographer Societies; EMICS, European Medical Imaging Coding System; EQF, European Qualifications Framework; ESR, European Society of Radiology; ESTRO, European Society for Radiotherapy and Oncology; EU, European Union; EURADOS, European Radiation Dosimetry Group; EURAMED, European Alliance for Medical Radiation Protection Research; HIS, Hospital Information System; IR, Interventional Radiology; IT, Information Technology; KPIs, Key Performance Indicators; KSC, Knowledge, Skills and Competence; LINAC, Linear Accelerator; MC, Monte Carlo; MEDRAPET; Medical Exposures Directive’s Requirements on Radiation Protection Training of Medical Professionals in the EU; MELODI, Multidisciplinary European Low Dose Initiative; MRI, Magnetic Resonance Imaging; NERIS, European Platform on Preparedness for Nuclear and Radiological Emergency Response and Recovery; NTCP, Normal Tissue Complication Probability; OPERRA, Open Project for European Radiation Research Area; PBPK, Physiologically-based Pharmacokinetic; PET, Positron Emission Tomography; PTV, Planning Target Volume; RIS, Radiology Information System; RSNA, Radiological Society of North America; RTD, Research and Technological Development; SPECT, Single Photon Emission Computed Tomography; SRA, Strategic Research Agenda; TCP, Tumour Control Probability
